# Synthesis of Polymerizable Cyclodextrin Derivatives for Use in Adhesion-Promoting Monomer Formulations

**DOI:** 10.6028/jres.114.001

**Published:** 2009-02-01

**Authors:** Rafael L. Bowen, Clifton M. Carey, Kathleen M. Flynn, Charles M. Guttman

**Affiliations:** Paffenbarger Research Center, American Dental Association Foundation, Gaithersburg, MD 20899-8546; National Institute of Standards and Technology, Gaithersburg, MD 20899-0001

**Keywords:** *beta*-cyclodextrin, dental materials, MALDI-TOF-MS, monomer synthesis, polymerizable cyclodextrin derivatives

## Abstract

The synthesis of the cyclodextrin derivatives reported herein was assisted by extensive literature research together with structure-property relationships derived from three-dimensional molecular modeling. These studies led to the hypothesis that many of the 21 hydroxyl groups on *beta*-cyclodextrin molecules could be derivatized to form a closely related family of analogous chemical compounds containing both polymerizable groups and hydrophilic ionizable ligand (substrate-binding) groups, each attached via hydrolytically-stable ether-linkages. The vinylbenzylether polymerizable groups should readily homopolymerize and also copolymerize with methacrylates. This could be highly useful for dental applications because substantially all contemporary dental resins and composites are based on methacrylate monomers. Due to hydrophilic ligands and residual hydroxyl groups, these cyclodextrin derivatives should penetrate hydrated layers of dentin and enamel to interact with collagen and tooth mineral. Analyses indicated that the diverse reaction products resulting from the method of synthesis reported herein should comprise a family of copolymerizable molecules that collectively contain about 30 different combinations of vinylbenzyl and hexanoate groups on the various molecules, with up to approximately seven of such groups combined on some of the molecules. Although the hypothesis was supported, and adhesive bonding to dentin is expected to be significantly improved by the use of these polymerizable cyclodextrin derivatives, other efforts are planned for improved synthetic methods to ensure that each of the reaction-product molecules will contain at least one copolymerizable moiety. The long-term objective is to enable stronger and more durable attachments of densely cross-linked polymers to hydrated hydrophilic substrates. Capabilities for bonding of hydrolytically stable polymers to dental and perhaps other hydrous biological tissues could provide widespread benefits.

## 1. Introduction

One of the challenges that the dental research community still faces is finding means for dentists to feasibly produce sufficiently strong and durable bonding of composites and resins to both dentin and enamel by physicochemical mechanisms that will not subsequently permit the formation of secondary (“recurrent”) caries or discoloration of tooth-resin interfaces. To accomplish this, both improved materials for adhesive bonding to dentin and enamel and proper clinical use of these materials are needed.

Monomers and comonomers should have sufficient solubility in water to penetrate, by countercurrent diffusion, into the hydration layers within the surfaces of dentin and enamel that have had all weak surface materials removed. These hygroscopic monomers should contain substantially no water when applied to these surfaces so they can take up and replace the water within these hydrated substrates. Also, the viscosity of such hygroscopic formulations should be sufficiently low to enable rapid infiltration and penetration of dentinal tubules and lateral canals without inducing aspiration of odontoblasts. Diluent comonomers for the polymerizable cyclodextrin derivatives should have similar solubility parameters to prevent phase separations during polymerization. Comonomers should be such as to result in a copolymer with high cross-link density. Polymerization initiators within the formulations must accompany the monomers during countercurrent diffusion into the hydrated substrates. Hydrophilic initiators with solubility parameters close to those of the hydrophilic monomers should be utilized to enable the polymerization initiator(s) to penetrate the hydration layers together with the monomers. An adequate concentration of monomeric components in the formulation should be able to form strong and numerous interfacial substrate-binding interactions per molecule sufficient to cover all of the accessible internal and external substrate surfaces.

The formulation should copolymerize into a densely cross-linked structure that will not be subject to hydrolytic or chemical degradation, and have higher adhesive strength than cohesive strength. The rationale for this last desired characteristic is that when margin gap formations occur in vivo due to polymerization or thermal shrinkage or other stresses, the gaps would expose two surfaces of cross-linked polymers rather than a space between polymer and dentin or enamel. Cariogenic bacteria can thrive so long as dentin or enamel can buffer the acids they excrete, but could not survive in a polymeric enclosure in which their acids would lower the pH to levels below their tolerance. A gap at the tooth surface would be an environment for bacterial growth and the sequel of secondary caries development. Therefore, the optimum is to have high cohesive strength of this hydrolytically stable copolymer and even higher adhesive strength at the copolymer-tooth interface.

The long term hypothesis to be tested is that polymers with high cross-link density containing no hydrolyzable ester (− C(= O)O − C −) groups will be more durable in dental applications than contemporary polymers that contain ester linkages upon which cross-linking and stability depend. The development of monomers that can form ester-free cross-linked copolymers will be the subject of the subsidiary work reported here. This report describes the preparation of a novel type of cross-linking monomers, which have both polymerizable groups and ionizable ligand (surface-binding) groups intramolecularly connected by means of hydrolytically stable ether (− C − O − C −) linkages, for use in polymerizable formulations for subsequent testing of the long-term goal.

Improvements have continued to evolve in the compositions of formulations for bonding composite restorative materials to dentin [1] and enamel since it was found that certain types of organic molecules could successfully compete with water for tooth surfaces [2,3]. Proper treatment for bonding dental resins to dentin and enamel includes an etching of the surfaces to remove weak surface material, such as adsorbed biofilms or, if instrumented, a fragmented and mechanically weak mixture of substrate materials [4]. This debris is also known to hinder or prevent the penetration and sealing of the dentinal tubules and their anastomotic lateral canals. The etching is usually done by application of a phosphoric acid solution or thixotropic gel, although acidic monomers are sometimes used. When phosphoric acid is used, the surface is rinsed with water to remove the solution or suspension of the weak surface debris and to open the dentinal tubules to permit their obturation by the adhesive bonding agents.

Thusly treated intertubular dentin surfaces comprise a hydrated layer of exposed collagen fibrils that are substantially attached to underlying mineralized dentin. These collagen fibrils, which remain covered with bound water molecules [5], are probably denatured to some degree. This moist demineralized protein and the underlying forms of calcium phosphate are the substrates to which adhesion must be achieved if good bonding is to be expected. In the case of enamel, the acid etching, rinsing, and blowing “dry,” results in a hydrophilic calcium phosphate surface in which the pores retain water.

The purpose of this report is to describe the synthesis of a family of copolymerizable molecules that collectively contained about 30 different combinations of vinylbenzyl and hexanoate groups on the various molecules, with up to approximately seven of such groups combined on some of the molecules. Such a family, we believe, can contain individual member molecules that can form higher concentrations of attractive interactions with complimentary “receptor sites” contained on and within cleaned dental substrates than can components of currently available adhesive bonding materials. This endeavor represents progress in the development of a relatively water-soluble family of molecules with most of the members containing both polymerizable groups, hydroxyl groups, and carboxylate-terminated ligand groups to form multiple electrostatic and ionic binding interactions with mineral-anchored collagen fibrils and the calcium phosphate phases of dentin and enamel.

Based on previous literature findings that described collagen fibrils [5–7] and beta-cyclodextrins [8–14], computer-assisted three-dimensional scale models of collagen segments and anticipated examples of *beta*-cyclodextrin’s polymerizable derivatives were prepared and studied. These computer simulations together with calculated estimates of structure-property relationships suggested that attempts to synthesize prototypic poly-merizable cyclodextrin derivatives as described herein could be of significant value.

The objective was to attach, by means of hydrolytically stable ether linkages, several polymerizable groups and hydrophilic carboxylate-terminated ligand groups per molecule of beta-cyclodextrin. Due to the hydrophilic ionic ligands and residual hydroxyl groups, novel family members are postulated to be able to penetrate the hydrated layers of dentin and enamel to interact with collagen and tooth mineral, and their poly-merizable vinylbenzyl groups to copolymerize with compatible diluent comonomers.

The hypothesis was that the synthesis procedure described herein would produce a family of diverse polymerizable cyclodextrin derivatives with many combinations and permutations of polymerizable and ionizable ligand groups attached by way of hydrolytically stable ether linkages. The rationale for a diversity of molecular configurations is that the quasi “receptor sites” in the substrates of interest are also very diverse. When a relatively anhydrous formulation containing these hydrophilic and hygroscopic polymerizable cyclodextrin derivative family members diffuses countercurrently into the hydrated tooth surfaces, there should be an enthalpy-promoted docking of the family members that have an appropriate number and configuration of ionic ligands and hydroxyl groups onto complementary receptor sites. An entropically favorable displacement of multiple substrate-bound water molecules should accompany these docking events.

It is posited that the water imbibed before, during and after polymerization will plasticize the resulting densely cross-linked adhesive layer, forming a thin, tough polymer that has high cohesive strength albeit lower than its adhesive strength. Adhesion test results, to be published in the following paper Ref. [1], will support this expectation.

Ether-mediated cross-linking of polymerizable cyclodextrin derivatives combined with diluent comonomers that also contribute to ether-mediated cross-linking are expected to provide long-term stability of their polymers under oral conditions. When optimal formulations and clinical applications of these monomers have been developed, adhesive polymers with improved hydrolytic stability are anticipated, relative to those obtained from monomers containing ester linkages as exist within contemporary adhesion-promoting formulations [15] and in the polymerizable cyclodextrin methacrylates that have been described previously [16–20].

## 2. Materials and Methods

### 2.1 Characteristics of *Beta*-Cyclodextrin

**Fig. 1:**
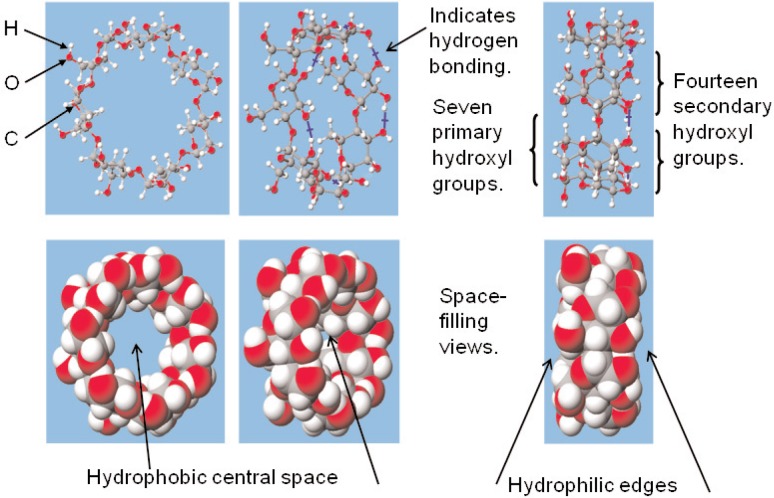
This shows ball-and-cylinder and space-filling images of modeled *beta*-cyclodextrin molecules; these molecules have internal cavity dimensions of about 0.7 nm, outside diameters of about 1.5 nm, and heights between the hydrophilic edges of about 0.7 nm.

*Beta*-cyclodextrin (BCD) is a cyclic oligosaccharide that consists of seven glucopyranose units, linked by alpha 1–4 glycoside linkages. The three-dimensional structure of a *beta*-CD molecule resembles a truncated cone with 14 secondary hydroxyl groups located on the edge of the larger opening and seven primary hydroxyl groups attached to the smaller opening of the cone. The interior of the cone is relatively hydrophobic. Structural characteristics of a modeled *beta*-cyclodextrin molecule are illustrated in Fig. 1.

### 2.2 Computer Modeling of *Beta*-Cyclodextrin, Reagents, Products and Formulations

Three-dimensional models of the components were studied with the use of a Computer-Aided Chemistry facility (CAChe 6.1.1 WorkSystem Pro including BioMedCAChe, Fujitsu Ltd., Beaverton, OR). This suite of computational chemistry applications operated on a 660 MHz dual processor PC with 1 GB RAM with a stereo 3D monitor.

### 2.3 Synthesis of Polymerizable Cyclodextrin Derivatives

The beta-cyclodextrin, received as a free sample from American Maize-Products Company, was crystallized by dissolving 27 g (as received) in 100 g of distilled water heated with stirring to 90 °C, letting the solution cool slowly to about 23 °C within an insulating chamber, and then decanting off the supernatant solution from the sparkling colorless crystals on the bottom. The decanted supernatant, which contained nonbirefringent micrometer-sized particulates that showed polarized light scattering from a laser pointer light beam, was discarded. The remaining wet *beta*-cyclo-dextrin crystals (23.07 g) were dried at 120 °C in a 30 MPa vacuum oven equipped with a slow flow of dried and filtered air.

Into a tared round bottom flask, equipped with a water bath and a mechanical stirrer, 5.0 g of the crystallized and dried *beta*-cyclodextrin (Table 1), 0.0345 g of Irganox 1330 (about 1 mole of this stabilizer per 100 mole of *beta*-cyclodextrin) and 12.37 g of calcium hydroxide were added. To this, 96.01 g of distilled water was added creating a translucent white suspension with a pH of about 13. However, as the temperature was raised to 60 ºC the solubility of the calcium hydroxide decreased and the pH decreased. Therefore, potassium hydroxide was added as necessary to obtain and maintain a pH of about 13. Then a mixture of 5.2633 g (about 15 moles per mole of *beta*-cyclodextrin) of 6-bromohexanoic acid (to attach the hydrophilic carboxylate ligands) and 4.3718 g (about 6 moles per mole of *beta*-cyclodextrin) of vinyl benzyl chloride (to provide the polymerizable groups) was added to the flask. The solution was maintained close to 60 °C for 4 d with continuous stirring.

### 2.4 Matrix Assisted Laser Desorption/Ionization-Time of Flight-Mass Spectrometry

MALDI-TOF-MS spectra of sampled reaction products were obtained intermittently during and after synthesis with use of a Bruker Reflex II mass spectrometer (Bruker Daltonics, Inc., Billerica, MA, USA) to provide the number and type of groups that became attached to the beta-cyclodextrin molecules. The methods for this analysis have been previously described [21]. Also, an analytical method, Polymerix (Sierra Analytics, Modesto, CA), was employed during this study to aid in peak identification.

### 2.5 Nuclear Magnetic Resonance Spectrometry

NMR spectra were obtained with the use of a JEOL GSX 270 MHz Fourier Transform Nuclear Magnetic Resonance Spectrometer (JEOL, Peabody, MA). 1H (proton, nondecoupled), 13C (carbon, decoupled) spectra, and 2-D heteronuclear correlations were obtained. The spectra were compared with available literature information depicting spectra of *beta*-cyclodextrin and some of its derivatives [22, 23].

## 3. Results

### 3.1 Characteristics of Beta-Cyclodextrin

After 24 h, and also after 4 d of drying, the mass of the crystallized beta-cyclodextrin was 19.37 g. A 5.0 g portion of these dried white crystals was used in the polymerizable cyclodextrin derivative synthesis.

### 3.2 Computer Modeling of Beta-Cyclodextrin, Reagents, Products and Formulations

**Fig. 2:**
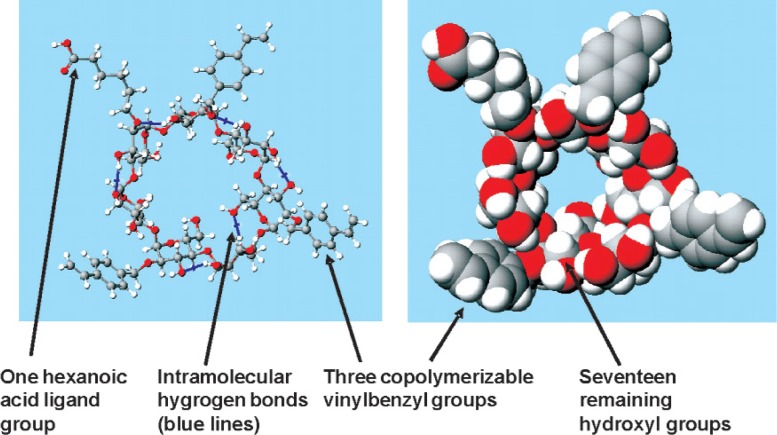
A ball-and-cylinder and a space-filling image of one of the many diverse reaction products of this polymerizable cyclodextrin derivative synthesis. This synthesis produced a family (group, ensemble, or collection) of copolymerizable cyclodextrin derivatives having many combinations and permutations; one family member is illustrated.

Three-dimensional modeling provided QSAR (quantitative structure property relationships) and predicted solubility (miscibility) characteristics that were useful in separating polymerizable cyclodextrin derivatives from unwanted byproducts and impurities. Figure 2 exemplifies one permutation of a polymerizable cyclodextrin derivative molecule that would be in accord with one of the MALDI spectral peaks.

### 3.3 Synthesis of Polymerizable Cyclodextrin Derivatives

This method of synthesis, which in retrospect was not considered an optimal method, did produce a family (group, ensemble, or collection) of products with cyclodextrin monomers containing up to approximately seven combined vinylbenzyl groups plus 6-hexanoate groups covalently attached by hydrolytically stable ether linkages to the various beta-cyclodextrin molecules. Therefore, there would remain 21 hydroxyl groups minus the number replaced by the copolymerizable and ligand groups on the various family members. These hydroxyl groups increased the predicted water solubility, in addition to that of the hydrophilic carboxylate-terminated ligand groups.

### 3.4 Matrix Assisted Laser Desorption/Ionization-Time of Flight-Mass Spectrometry

**Fig. 3:**
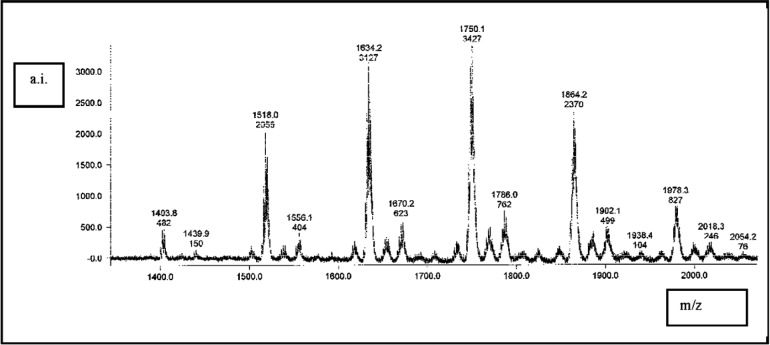
A MALDI-TOF MS spectrum of about 30 diverse reaction products obtained by the method of synthesis reported herein that produced a family of copolymerizable molecules. The upper numbers above some of the peaks signify mass per positive charge values (m/z), and the numbers just beneath those represent the relative abundance of their detected ions (a.i.). For clarity, only 14 of the peaks are shown with these numbers. The peaks on the left side of the spectrum correspond to *beta*-cyclodextrin derivatives containing fewer vinylbenzyl and hexanoate groups per molecule, whereas peaks toward the right side indicate the *beta*-cyclodextrins containing larger numbers of vinylbenzyl and hexanoate sub-stituents.

MALDI-TOF-MS was very useful in identifying many of the derivatives, the output peaks of which were labeled with calculated mass values and an indication of the abundance of the detected molecular ions (a.i.) that carried one positive charge. Internal calibration standards were not used in these experiments, therefore prior external calibration for mass to charge was performed as described previously [21]. The synthesized reaction products (polymerizable cyclodextrin derivatives) contained many different combinations of both vinylbenzyl polymerizable groups and potassium hexanoate ligand groups combined on individual family members. Adding to the diversity of spatial configurations of such a family of polymerizable cyclodextrin derivatives would be the various permutations (positional arrangements of vinylbenzyl and hexanoate groups on the 21 potential sites) within these combinations. Figure 3 shows an abridged indication of the spectral peaks obtained. The Figure 3 spectrum and the results given in Table 2 were obtained by the Polymerix analytical method.

### 3.5 Nuclear Magnetic Resonance Spectrometry

The many NMR spectra obtained with the starting reagents and reaction products were all in accord with relevant information obtained from reference texts, with literature relating to *beta*-cyclodextrins, and with the MALDI analytic findings.

## 4. Discussion

Based on these studies, *optimized* polymerizable cyclodextrin derivative-containing formulations are predicted to be good adhesion-promoting bonding agents. Such formulations would contain diverse hetero-multifunctional polymerizable cyclodextrin derivatives containing combinations and permutations of ligands, attached via hydrolytically stable ether linkages, to chemically interact with receptor sites of both the organic and inorganic components of hard tooth tissues and polymerizable vinylbenzylether groups to copolymerize with the monomers of dental resins and composites. It can be speculated that optimization might be approached by adjusting the relative quantities and ratios of the reagents that attach ligand and polymerizable substituents to the cyclo-dextrin scaffolds, and influencing the permutations of these substituents by means of appropriately adjusting the pH values of the reaction medium during the sequential addition of these reagents.

There is literature indicating that surface-active comonomers (SACs) with multiple ligand groups, together with polymerizable groups, are effective for adhesive bonding of resins to teeth [2–3, 16, 24, 25]. However, in the thousands of references to cyclodextrins and their derivatives in the general literature (e.g., [8, 9, 10, 13, 14, 23, 26–28]), those found in extensive searching were not used as dental materials.

The synthesized family of reaction products described in this report is expected to have a significant portion of its polymerizable cyclodextrin derivatives contain multiple polar groups to form simultaneous enthalpic and entropic interactions to enable replacement of water on “receptor sites” of the substrates, and also contain cross-link-forming monomeric components devoid of ester groups connecting their two or more polymerizable groups to void the potential for ester linkages to become unlinked by hydrolysis or saponification in oral environments [29]. Polymerizable cyclodextrin derivative monomers like these [30], together with diluent comonomers and photo initiators as exemplified elsewhere [31], can reasonably be expected to provide good penetration and high concentrations of intermolecular attractive interactions with the components of etched dentin.

Bulky hydrophobic initiators might be partitioned outward during diffusion into deeper hydrated regions, unless inactive portions of their molecules, by partial or reversible complexation within the hydrophobic “host” spaces of outwardly hydrophilic polymerizable cyclodextrin derivatives can convey such initiators to wherever the monomers migrate. Smaller hydrophobic initiators such as camphorquinone and ethyl 4-(dimethylamino)benzoate (“4E”), which must come into physical contact to form radical-producing exiplexes, are thought to become sterically hindered if complexed within separate hydrophobic “host” spaces of polymerizable cyclodextrin derivatives [18].

## 5. Conclusions

This prototypic synthetic procedure succeeded in supporting the hypothesis that *beta*-cyclodextrin could be derivatized to form a family of monomers having both polymerizable and ionizable ligand groups attached by means of ether linkages. It is well known that ether linkages are much more hydrolytically stable than are ester linkages, which is especially important in linkages within cross-linked polymers. The synthetic method described herein derivatized up to approximately seven of the 21 hydroxyl groups on these cyclodextrin molecules with polymerizable and/or ligand groups, as shown by analysis of the spectra obtained with matrix assisted laser desorption/ionization-time of flight-mass spectrometry (Table 2). The work reported here together with an accompanying report “Adhesive bonding to dentin improved by poly-merizable cyclodextrin derivatives” indicate a potential for formulations containing polymerizable cyclodextrin derivatives to provide for significantly improved adhesive bonding interactions of cross-linked polymers with hydrous biological tissues.

## Figures and Tables

**Table 1:** Materials used in the synthesis of a family of polymerizable cyclodextrin derivatives

Acronym	Chemical	Lot	Manufacturer
BCD	beta-cyclodextrin	G 6020-42 USP	American Maize-Products Co. (now Cerestar USA, Inc.,) Hammond, IN 46320
Irganox 1330	1,3,5-trimethyl–2,4,6-tris(3,5-di-(tert)-butyl–4-hydroxybenzyl)benzene	11107	Ciba Specialty Chemicals Corp., Tarrytown, NY 10591
6-BHA	6-bromohexanoic acid	17519JO	Sigma-Aldrich, Inc., St. Louis, MO 63103
VBC	vinylbenzyl chloride	MZ 05522EZ	Aldrich Chemical Co., Inc., Milwaukee, WI 53233

**Table 2:** An illustrative sampling of vinylbenzyl polymerizable groups and of potassium hexanoate ligand (surface-binding) groups obtained by analysis of peaks shown in the MALDI-TOF MS spectrum of Fig. 3

BCD Substituents		
Vinybenzyl	K Hexanoate	(% of each kind)
1	1	1
1	2	2
1	3	3
1	4	3
1	5	2
1	6	1
2	1	1
2	2	1
2	3	1
2	4	1
2	5	1
3	2	1
3	3	1
4	0	2
4	1	2
4	2	1
4	3	1
5	0	1
5	1	1
